# Bone implant sockets made using three different procedures: a stability study in dogs

**DOI:** 10.4317/jced.50803

**Published:** 2012-10-01

**Authors:** Jorge Cano, Julián Campo

**Affiliations:** 1 DDS, MSc, PhD, Lecturer. Department of Buccofacial Medicine and Surgery. School of Dentistry, Complutense University of Madrid. Spain.; 2 DDS, PhD, Lecturer. Department of Buccofacial Medicine and Surgery. School of Dentistry, Complutense University of Madrid. Spain.

## Abstract

Objective: This study compared the effects of three different methods of preparing bone implant sockets (drilling, osteotomes, and piezoelectric device) on osseointegration using resonance frequency analysis (RFA). Study Design: An experimental prospective study was designed. Material and Methods: Ten adult beagle dogs were studied. After 5 weeks, 23 out of 28 initially placed implants in the iliac crest were evaluated, comparing these three different procedures of bone implant socket. Student’s t-test (paired, two-tailed) was used to reveal differences among the three groups at each time point (SPSS 16.0, IL, USA). Results: After a 5-week healing period, the implants placed in sockets that were made using an osteotome or piezoelectric device were slightly more stable than those made by drilling. Reduced mechanical and heat injury to the bone is beneficial for maintaining and improving stability during the critical early healing period. Conclusion: Using RFA, there was evidence of a slight increase in implant stability in the iliac crest after 5 weeks of healing when the implant socket was made using a piezoelectric device or expansion procedure as compare with the drilling method.

** Key words:**Bone implant sockets, drilling, osteotomes, piezoelectric, resonance frequency analysis, stability.

## Introduction

Albrektsson et al. (1) described some of the factors that are critical for achieving predictable osseointegration around implants. One is the surgical technique used to make the bone implant socket. The cortical bone in the most coronal area of the alveolar ridge is critical in bone preparation. Consequently, new implant insertion techniques attempt to preserve cortical bone and condense the trabecular bone (2). Bone preparation by drilling results in an area of bone necrosis directly proportional to the heat produced by the burs. With normal drilling at 2000 rpm, the necrosis extends 1 mm. This area must be remodeled with new bone before the implant can be loaded (3,4).

Primary stability is the absence of mobility in the bone bed upon the insertion of the implant and depends on the quantity and quality of bone, surgical technique and implant design. Secondary stability depends on bone formation and remodeling at the implant-bone interface and is influenced by the implant surface and the wound healing time, that is activated after the surgical injury produced during preparation of the implant site (5,6).

Few studies have been made on the outcome of osseointegration of alveolar bone around dental implants inserted with piezoelectric osteotomy or osteotomes versus conventional osteotomy by burs (7,8).

Resonance frequency analysis (RFA) is an objective, reliable, non-invasive method used to assess bone-implant interface stability. RFA provides a meaningful clinical index for the early assessment of the quality of the implant-bone interface (primary stability) and secondary stability after healing (7,9).

In 1998, Meredith et al. (10) published a study on non-invasive techniques and their application for measuring endo-osseous implant stability and osseointegration.

The goal of this experimental prospective study was to evaluate three different techniques for preparing the implant bone socket. Our null hypothesis was that bone preparation using a piezoelectric knife would result in better stability at 5 weeks as compared with unloaded implants.

## Material and Methods

1.- Animals: The study used 10 male beagle dogs (age ~2 years, weight 10~15 kg). The study was approved by the ethics committee for animal experimentation of Gomez Ulla Central Military Hospital (Madrid, Spain). For implant placement, anesthesia was induced with an intramuscular injection of medetomidine (20–40 mg/kg; Domtor®, Pfizer, Madrid, Spain) and butorphanol (0.2–0.4 ml/kg; Torbugesic®, Fort Dodge Veterinaria, Gerona, Spain). The dogs were intubated and anesthesia was maintained with 1.5–2% isoflurane and 60% NO2 and 40% O2 at a tidal volume of 12 ml/kg. The care and use of the experimental animals complied with local animal welfare laws, guidelines, and policies.

Three implants were placed in the iliac crest of each animal. Each bone implant socket was made using a different procedure. The posterior, central, and anterior sockets were made by drilling using burs, osteotomes, and a piezoelectric device, respectively. RFA was performed at the initial intervention and 5 weeks later. For each animal, three measurements were made. The results were grouped by socket preparation method: Group I, sockets made by drilling; Group II, sockets made with osteotomes; and Group III, sockets made with a piezoelectric knife.

**Figure 1:**
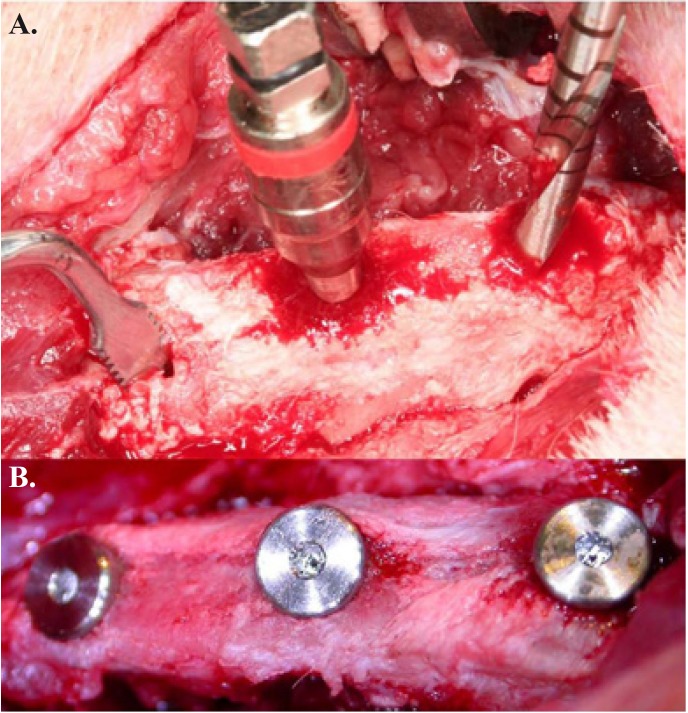
a.- Bone sockets made using three different procedures: drilling (right), osteotomes (center), and a piezoelectric device (left); b.- Photographs of the implants in place with cover screws.

**Figure 2:**
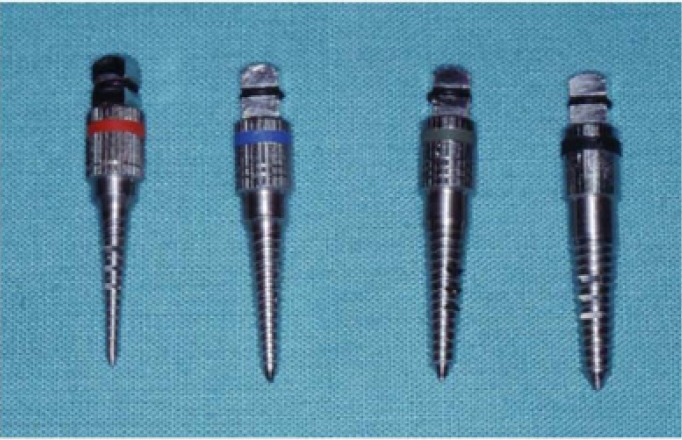
Threaded osteotomes.

2. Surgery: All interventions were performed in an animal operating room under sterile conditions. A 10-mm-long semilunar incision was made after shaving the hair and disinfecting the pelvic area (10 cm lateral and cranial to tail the insertion). The subcutaneous tissues and muscle insertions were detached, exposing the iliac crest. Three marks were made with a round bur, 10 mm apart. The first socket was made in the posterior-most position using an incremental sequence of burs (2, 2.8, and 3.2 mm in diameter). The second socket was made by first using a 2-mm bur and then a bone condensation sequence with threaded osteotomes (Micro-dent, Barcelona, Spain) (Fig. 1) until the socket was 3.2 mm wide.. An implant measuring 3.75 mm wide and 10 mm long (acid-etched surface, Bioner, Barcelona, Spain) was placed in each socket (Fig. 1). The last socket was prepared with a piezoelectric device (Surgysonic, Esacrom, Bologna, Italy) (50 Watts and 24~32 Hz) (Fig. 2). The wound was then closed in two layers using interrupted mattress sutures with 3-0 resorbable Vicryl (Et-hicon, Somerville, USA). For the next 3 days, all animals were given intramuscular streptomycin + penicillin G (2 ml/animal/day; Vetione®, Schering-Plough, Segre, France) and flunixin (1.1 mg/kg/day; Finadyne®, Schering-Plough, Segre, France).

**Figure 3:**
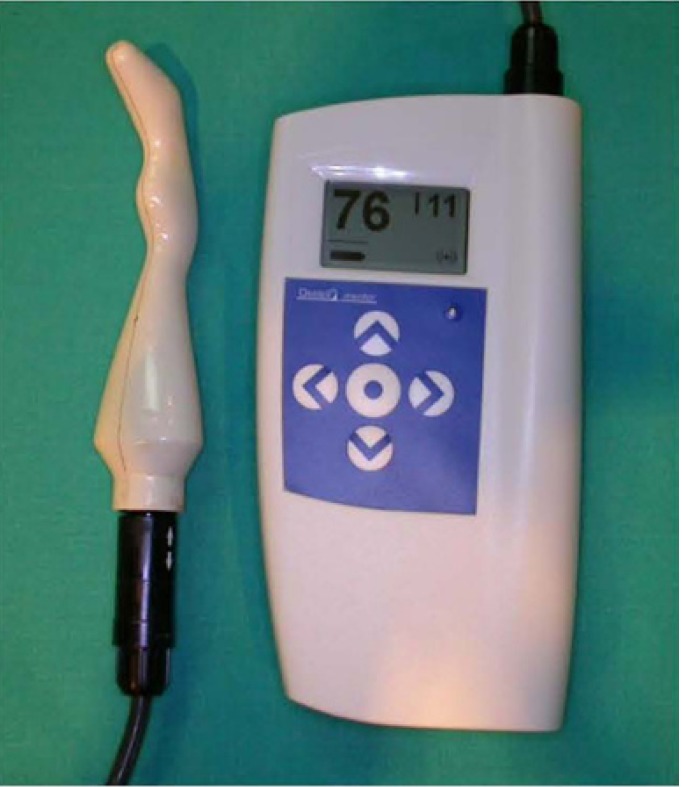
An Osstell transducer.

3.-Resonance Frequency Analysis: An Osstell frequency resonator was used (Integration Diagnostics, Goteborg, Sweden) (Fig. 3) with a 5-mm-high transducer abutment compatible with an external universal hexagon plat-form. Measurements were made after the initial implant placement and 5 weeks later. The implants were evaluated using the Implant Stability Quotient (ISQ), which is a scale from 1 to 100.

4. Statistical analysis: A Student’s t-test (paired, two-tailed) was used to reveal differences among the three groups at each time point (SPSS 16.0, IL, USA). The level of statistical significance was set at p < 0.05.

## Results

All 10 animals recovered well from the surgery and feeding and general health were good throughout the experiment. Owing to the anatomy of the iliac crest, only 28 implants were placed initially: 9, 10, and 9 in groups I, II, and III, respectively. In the subsequent 5 weeks, 5 implants were lost and 23 remained (8, 7, and 8 in groups I, II, and III, respectively). The failure percentage was 11.1, 30, and 11.1% in groups I, II, and III, respectively.

 Table 1 summarizes the RFA measurements. Initially, the ISQ was higher in the drilling group (p=0.108). After 5 weeks, the stability was reduced in the drilling group and increased in groups II and III (p=0.763).

**Table 1:**
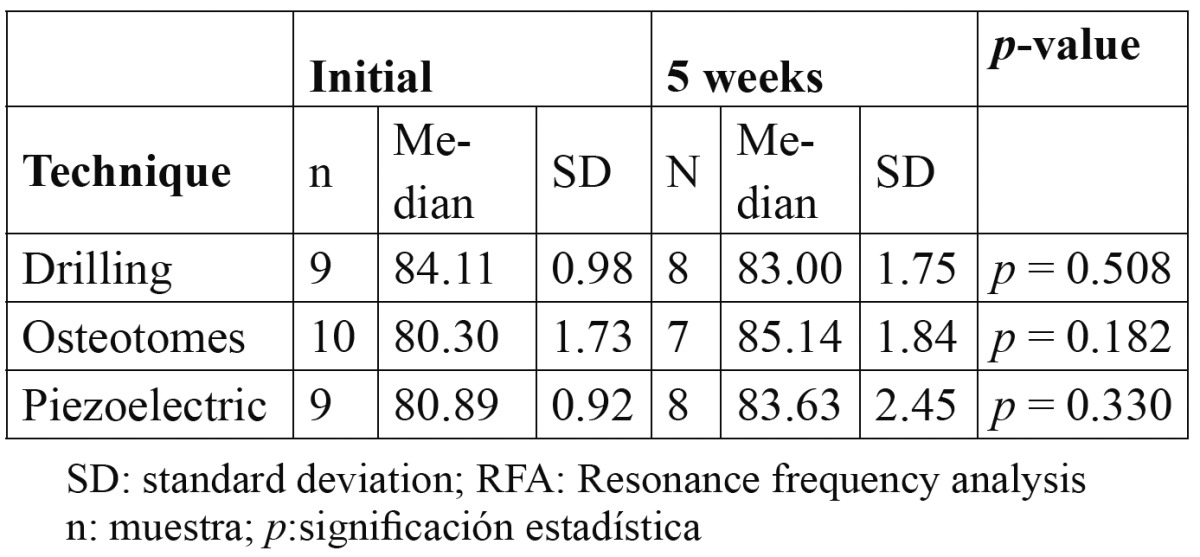
RFA measurements at initial and 5 weeks with the 3 different bone sockets preparations.

## Discussion

The beagle iliac crest is a good biomodel for evaluating bone implant sockets. The width and height of the crest are sufficient for placing standard dental implants without complications. The beagle mandible has been used as a biomodel for bone condensation (11); however, it requires a previous horizontal resection and has insufficient trabecular bone volume.

The drilling technique provided better initial stability in our study. This is a more precise procedure for making bone sockets of a specific size. The other two techniques might result in a greater discrepancy between the implant surface and adjacent bone. By contrast, after 5 weeks, the ISQ had increased with the osteotome and piezoelectric procedures, which do not produce heat. Bone heating causes increased necrosis, which reduces the secondary stability during the initial stage of healing.

Di Alberti et al. (7) demonstrated that piezoelectric implant site preparation promotes better bone density and osteogenesis compared with traditional surgical technique in forty patients. The bone density was studied with a densitometry application on radiographs taken at 30, 60 and 90 days. They concluded that the piezoelectric technique is predictable with a 100% success rate in this study.

The importance of bone expansion with bone condensation is misleading. Condensation of trabecular bone is used in order to increase the primary stability of the implants. Bone expansion includes the condensed bone and increased alveolar ridge width. Long-term follow-up studies report a 97% implant success rate using the expansion technique (12).

Krafft et al. (8) made an in vitro study using osteotomes for implant bed preparation compared with drilling. Bone quality was assessed by measuring implant insertion torque and primary implant stability by RFA, and found that the application of osteotomes leads to a significant higher values of RFA and implant insertion torque compared with implant sockets by means of drilling.

In a recent review RFA, as a technique for measuring dental implant stability has attracted considerable scientific interest in recent years, due to quantitatively and qualitatively properties to analyze the stability of various types of implants, surfaces, implant site preparations and to examine their behavior under different bone loading conditions (6).

Using RFA in our study, there was evidence of a slight increase in implant stability in the iliac crest after 5 weeks of healing when the implant socket was made using a piezoelectric device or expansion procedure as compare with the drilling method. However, the difference was not statistically significant. Further studies should compare the stability results with histological features.
